# Resective and Regenerative Approach for an Unresolved Periapical Lesion: A Surgical Case Report With 24-Month Follow-Up

**DOI:** 10.7759/cureus.49717

**Published:** 2023-11-30

**Authors:** Anchu R Thomas, Melwin Mathew, Sunil K Nettemu, Anoop Mayya

**Affiliations:** 1 Endodontics, Manipal University College Malaysia, Melaka, MYS; 2 Dentistry/Periodontics, Manipal University College Malaysia, Melaka, MYS; 3 Prosthodontics, Manipal University College Malaysia, Melaka, MYS

**Keywords:** gtr, bone graft, periapical surgery, mta, root canal

## Abstract

The pulp and the periodontium are inherently interconnected, both anatomically and functionally. Conditions affecting the periodontium and the pulp can create challenges in diagnosis, treatment strategizing, and predicting outcomes. This case report outlines the combination of resection and regeneration techniques utilizing mineral trioxide aggregate (MTA) and bone grafting to address a persistent periapical lesion in a maxillary premolar. The treatment led to the effective alleviation of the patient's symptoms and successful regeneration.

## Introduction

The predominant goal of all endodontic treatments, particularly cleaning and shaping, is to remove necrotic tissue and infective bacteria. Initially, non-surgical treatment is recommended for large periapical lesions of inflammatory origin [1]. The prevalence of persistent apical periodontitis after conventional root canal treatment among the adult populations in various countries ranges between 27%-70% and increases with age [2].

Periapical lesions represent a frequent pathological occurrence that affects the tissues surrounding the root apex of a tooth [3]. The invasion of microorganisms and subsequent infection within the root canal systems significantly influence the onset and advancement of periapical lesions [4].

In periapical lesions of endodontic origin, the occurrence of microorganisms is 87%, with viable and active microorganisms present in 82% of these lesions. The predominant bacterial species identified in these periapical lesions are Actinomyces, Fusobacterium, and Prevotella [5]. Actinomyces stands out as one of the most detected bacterial species in periapical lesions. This could be attributed to its ability to resist phagocytosis, which is facilitated by its capacity to aggregate among its own species [6]. Fusobacterium is another genus commonly detected, consisting of gram-negative anaerobic bacteria that possess the capability to adhere to, invade, and survive within both epithelial and endothelial human cells [7].

Surgical intervention should only be considered when nonsurgical endodontic therapy techniques have proven ineffective in resolving infection and periapical disease [4]. Surgical endodontics includes root resection, retrograde preparation of the canal using burs or ultrasonic tips, and retrograde restoration using biocompatible materials like MTA [8]. Research has shown that using a regenerative approach along with surgical endodontics toward periapical healing improves the outcome of bony lesions compared to the same lesions without a regenerative approach [9].

This case report discusses an unresolved periapical lesion that was successfully treated with surgical endodontics along with regeneration of the defect site.

## Case presentation

In May 2019, a 40-year-old male presented to the Department of Endodontics at Manipal University College Malaysia, Melaka, Malaysia, with a chief complaint of dull pain and discomfort in his upper root canal treated right second premolar, which worsened during mastication. The root canal treatment was done two years ago. The patient’s medical history was non-contributory. On intraoral examination, #15 had a fractured buccal cusp, a sinus tract opening, and tenderness on percussion. On radiographic examination, a periapical lesion measuring approximately 1x1.5 mm was observed, accompanied by periodontal ligament widening and mild root resorption. According to the clinical and radiographic findings, re-treatment of #15 was advised, and coverage of Miller’s Class 1 gingival recession present in relation to #14 was planned. Unfortunately, the patient did not turn up for further treatment during the COVID-19 outbreak due to the nationwide lockdown and concern over COVID-19 transmission.

**Figure 1:**
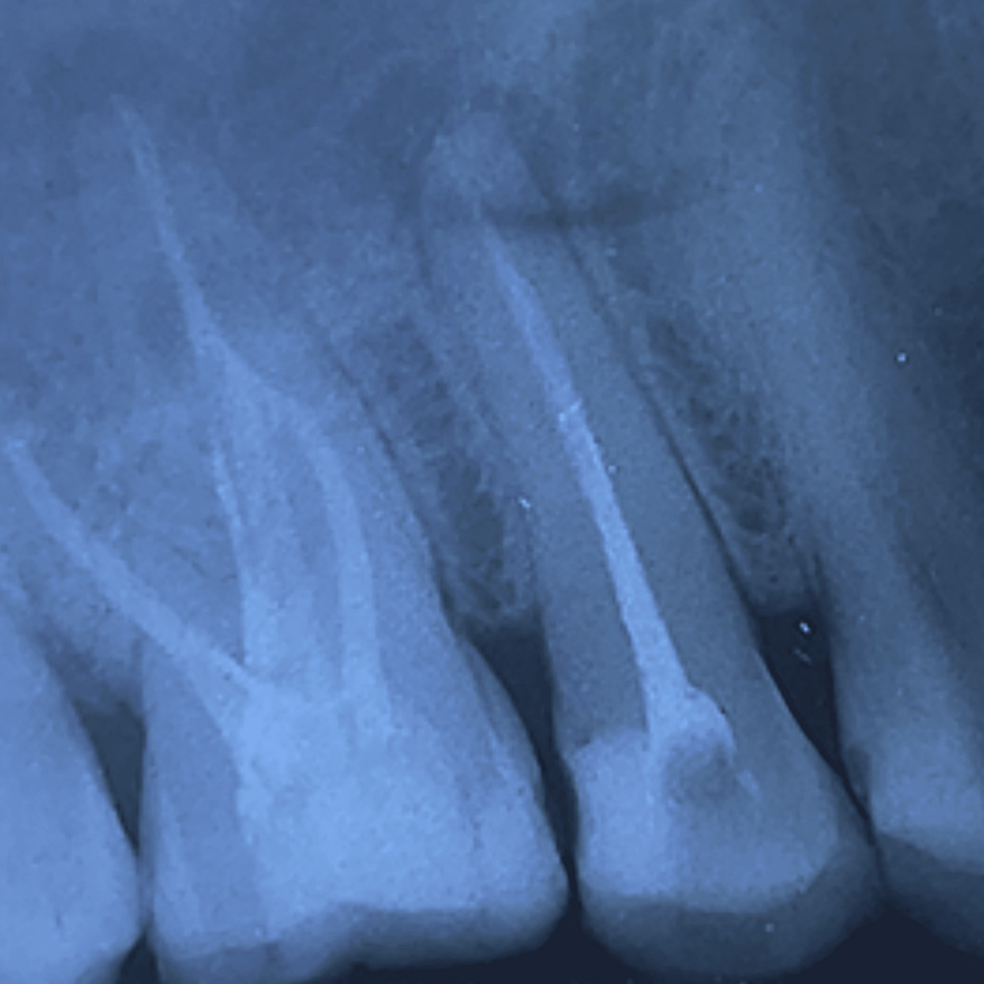
Preoperative IOPA with composite build-up of #15 IOPA: intraoral periapical radiography

After 14 months, the patient experienced severe pain on mastication in the same tooth, prompting the initiation of emergency re-treatment. There were no other new clinical signs. Vertucci’s type II canal morphology was observed in the pre-operative radiograph. A composite build-up of the lost tooth structure was done to facilitate rubber dam placement (Figure 1).

Access was gained to the canals using an endo access bur (Dentsply Sirona Inc., Tulsa, Oklahoma). The gutta-percha removal was carried out by GP solvent (Endosolv E, Septodont Holding, Canada) and ProTaper Universal System retreatment files (PTUS, Dentsply Sirona Inc., Tulsa, Oklahoma).

A drop of solvent was placed in the chamber to soften the gutta-percha; a total of 0.5 mL of solvent was used during the retreatment procedure for each canal. PTUS instruments D1, D2, and D3 were used for retreatment in the crown-down technique until D3. The working length was re-determined using the apex locator (Root ZX, J. Morita Corp., Tokyo, Japan). Each canal was prepared with ProTaper Next rotary files, X1 till X2 (Dentsply Maillefer, Ballaigues, Switzerland). During instrumentation, the root canal was irrigated with 6 ml of 5.25% NaOCl, followed by normal saline. The irrigant was activated with passive ultrasonic irrigation using passive ultrasonic tips (Irrisafe # 20/02, Newtron, Acteon, United Kingdom) for 30 seconds each. Then, calcium hydroxide intracanal medicament (Ultracal XS, Utradent, Jordan) was placed, and the access preparations were sealed with a temporary filling material (Coltsol, Coltene, Altstatten, Switzerland).

**Figure 2:**
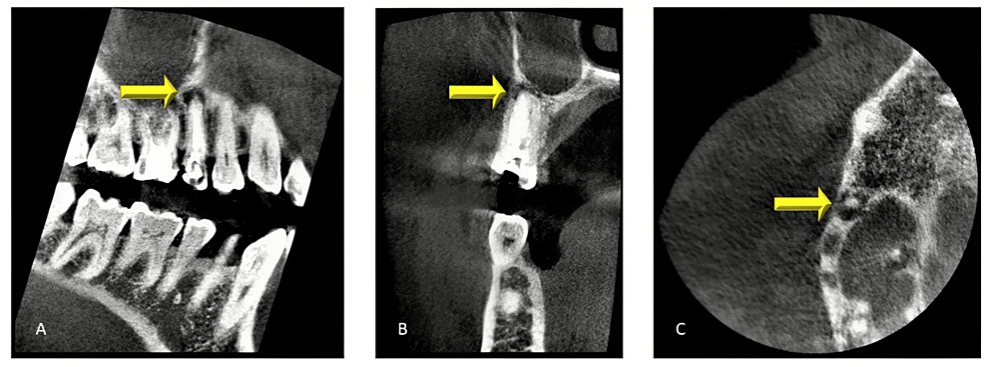
A sectional CBCT image shows a periapical lesion about 1.8x2.4 mm in size in relation to #15, along with mild root resorption. A) Sagittal view; B) Coronal view; C) Axial view CBCT: cone-beam computed tomography system

Despite several visits of root canal irrigation and activation with 5.25% sodium hypochlorite followed by calcium hydroxide intracanal medicament placement, the pain on mastication did not subside. After eight months of initiating the re-treatment, the pain persisted in the periapical region on mastication. Subsequently, a sectional cone-beam computed tomography system (CBCT) (Planmeca Promax 3D, Planmeca Oy, Helsinki, Finland) was advised. CBCT revealed a periapical lesion approximately (1.8 x 2.4mm) in relation to #15, approximating the maxillary sinus floor (Figure 2).

Based on the CBCT findings and the patient’s symptoms, surgical intervention following obturation was planned with the patient’s consent.

**Figure 3:**
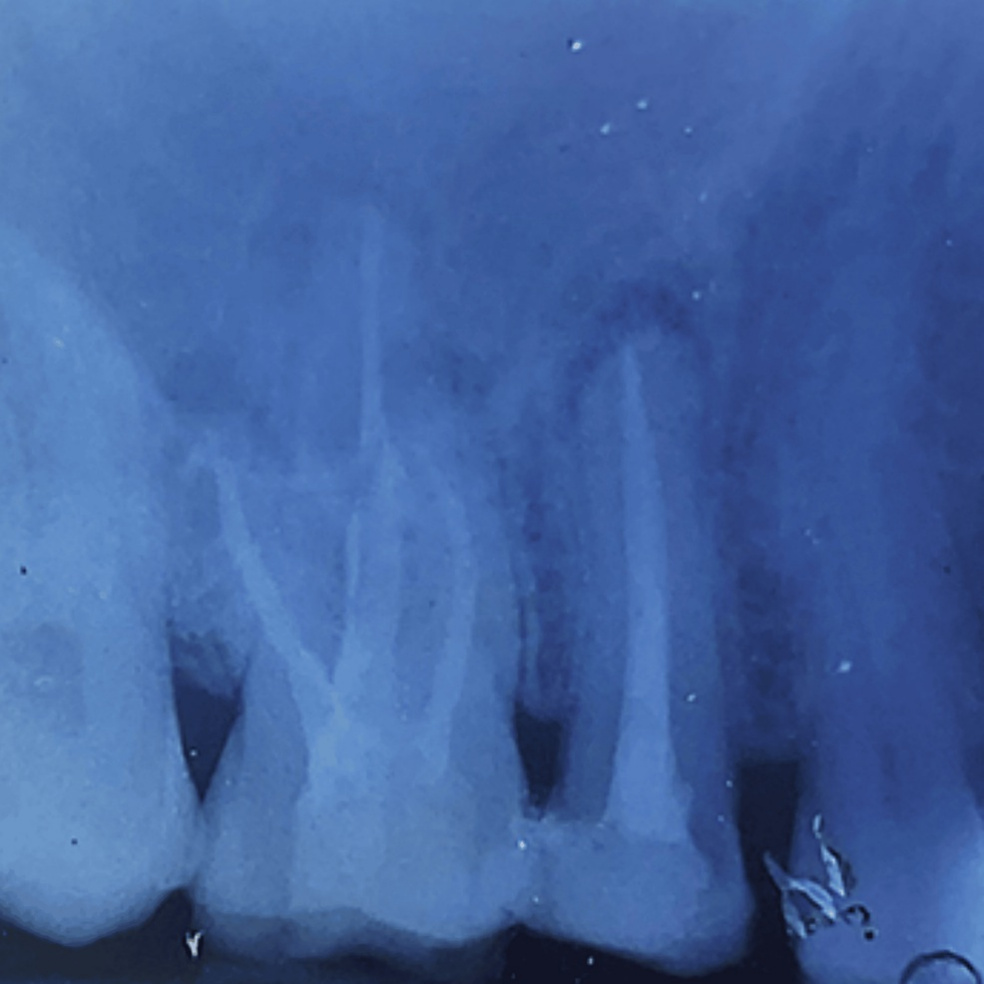
Final obturation of #15

Single cone obturation was performed using the ProTaper Gutta-Percha (Dentsply Maillefer, Ballaigues, Switzerland) and AH plus sealer (Dentsply DeTrey GmBH, Konstanz, Germany) (Figure 3).

The coronal access was restored with an RMGIC base (Vitremer, 3M ESPE AG, Seefeld, Germany) and composite restoration (Tetric Ceram, Ivoclar Vivadent Marketing (India) Pvt. Ltd., Gurugram, India).

**Figure 4:**
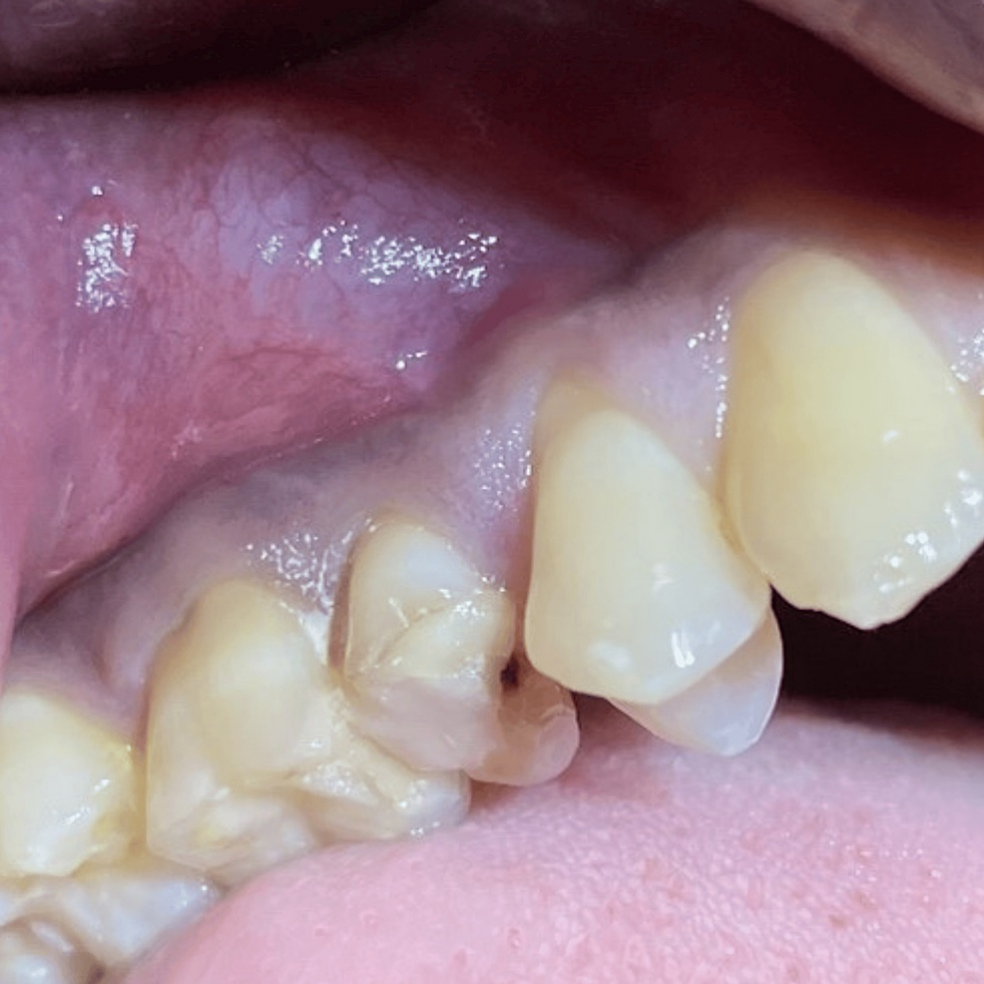
Pre-operative image: gingival recession seen in #14

Periapical surgery was performed under local anesthesia with 3% mepivacaine hydrochloride and epinephrine 1:100,000 (Scandonest 3%, Septodont, Saint-Maur-des-Fosses, France) (Figure 4).

A papilla preservation flap was reflected in relation to #14, #15, and #16 with a vertical incision in the mesial region with intent for gingival coverage for the recession in relation to #14. A lateral window was created in relation to #15 using a round surgical bur (QS Dental Supply Sdn Bhd, Kuala Lumpur, Malaysia). Cautious debridement of the region with a surgical curette was carried out to prevent accidental penetration into the maxillary sinus floor.

**Figure 5:**
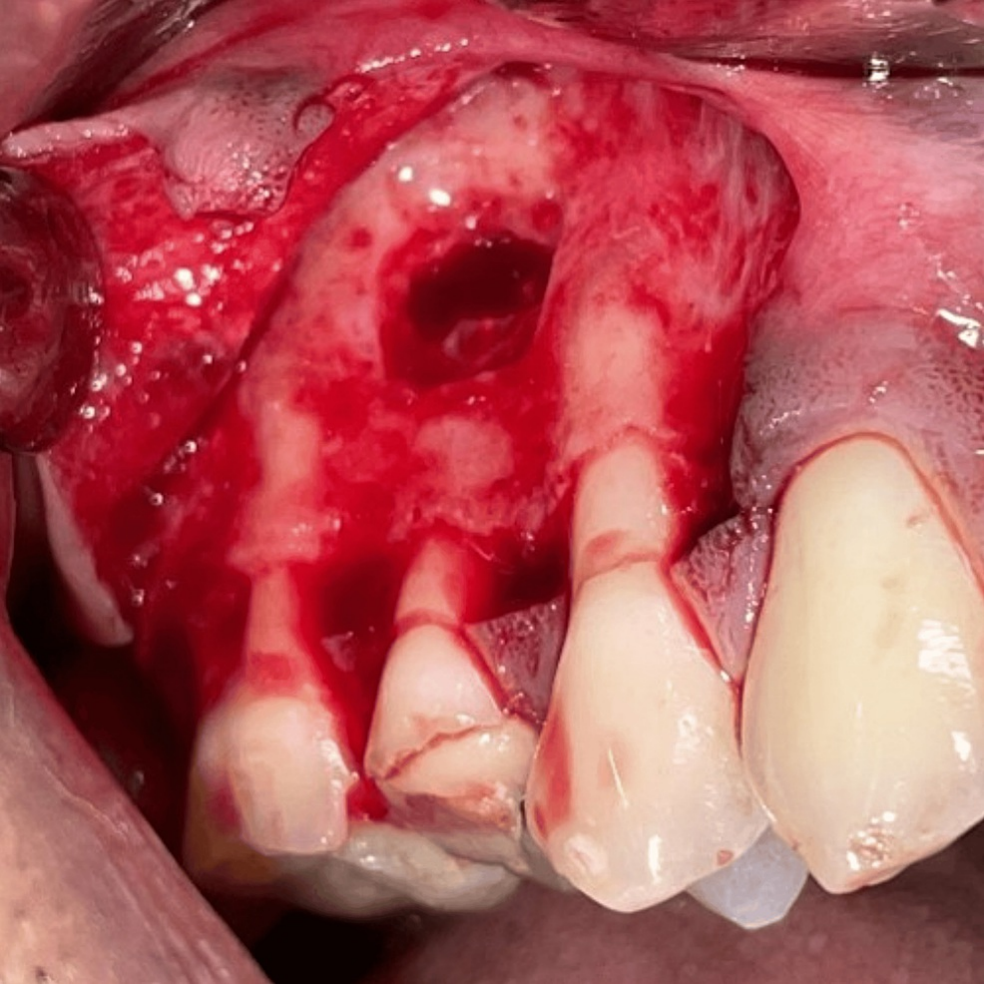
The papilla preservation flap is raised, and lateral window preparation is done for access to the periapical region of #15, followed by root end resection

**Figure 6:**
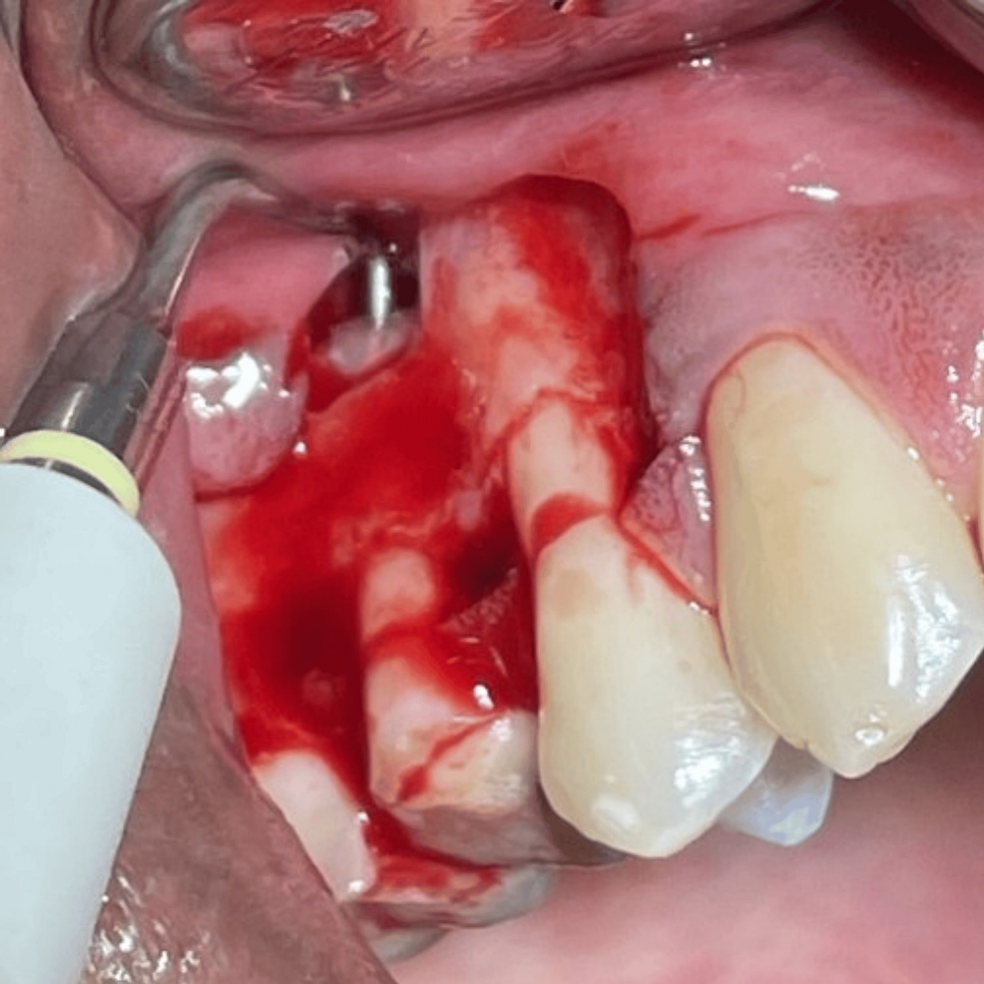
Root-end cavity preparation with ultrasonic tips

**Figure 7:**
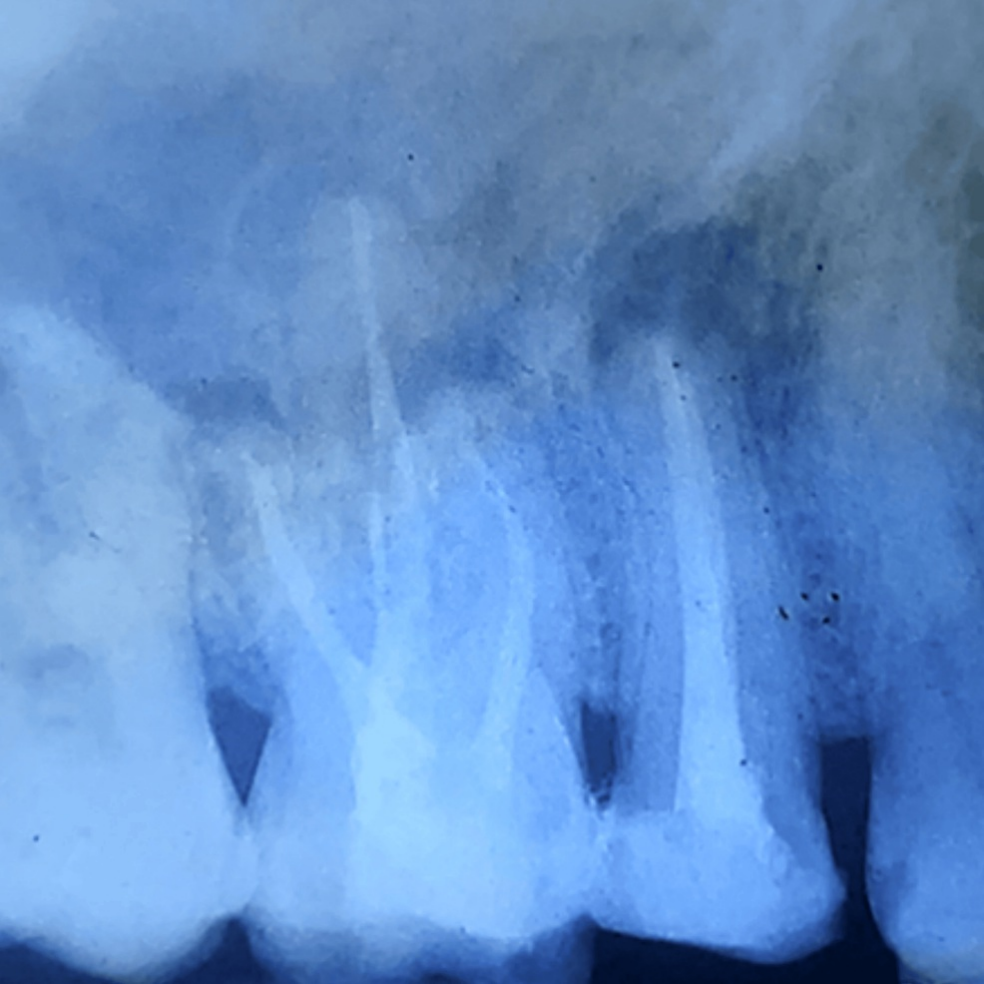
IOPA after debridement and root end resection of #15 IOPA: intraoral periapical radiography

On complete debridement of the infected tissues from the periapical site of #15, a root-end resection of 3mm at a shallow 0-10° bevel was done carefully using a 45° surgical handpiece (NSK Asia, Singapore) and Lindemann bone cutting bur (Strauss Diamond Instruments Inc., Florida, United States) with a resection angle perpendicular to the long axis of the tooth (Figure 5), followed by root-end cavity preparation with Start X Ultrasonic tips (Dentsply Sirona Inc., Petaling Jaya, Selangor, Malaysia) in both roots (Figures 6, 7).

**Figure 8:**
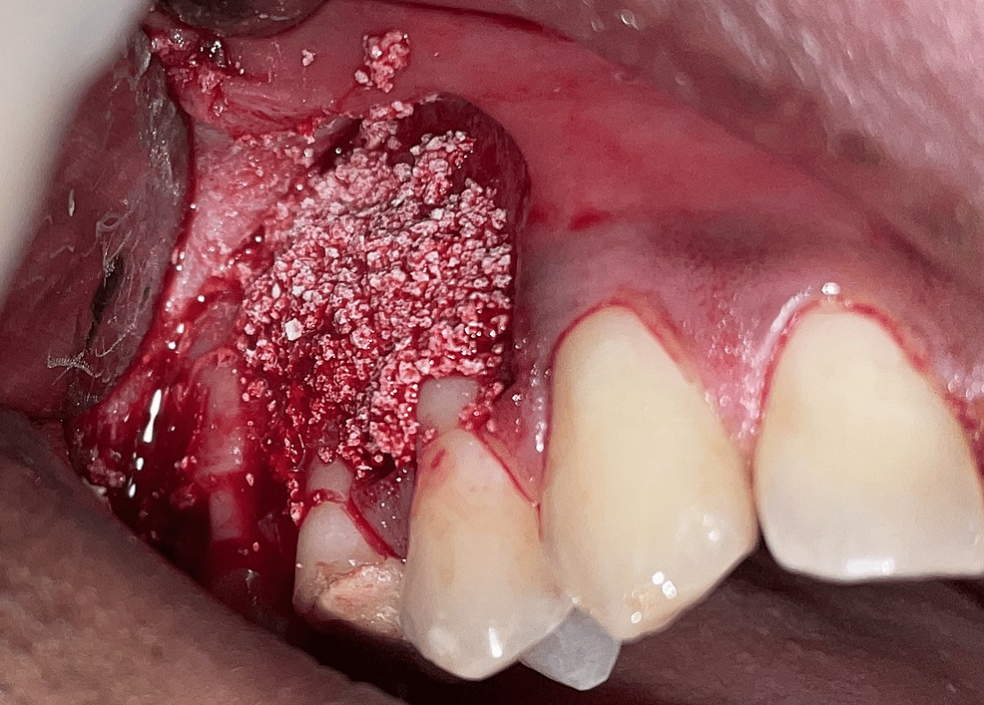
Bone graft placed in the periapical region of #15

**Figure 9:**
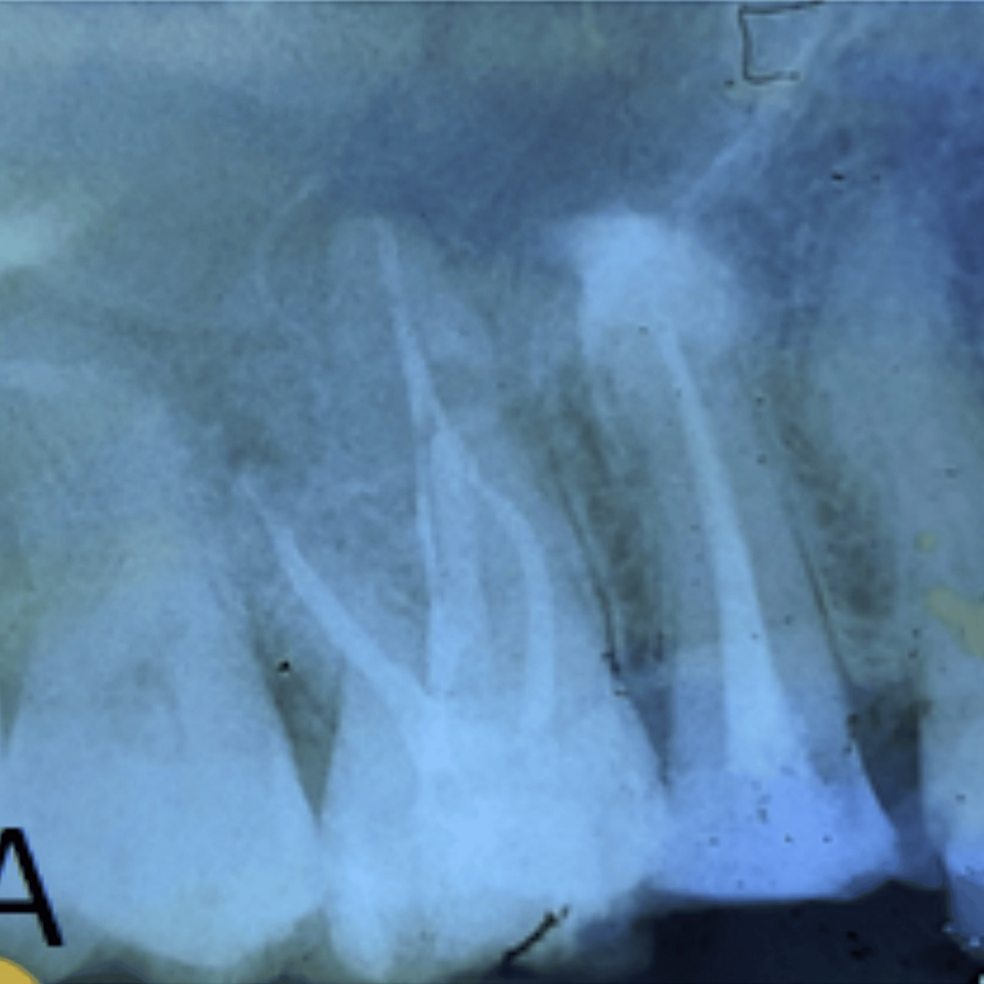
IOPA after MTA and bone graft placement in #15 IOPA: intraoral periapical radiography; MTA: mineral trioxide aggregate

The fine isthmus area at the root end was debrided using the tips, and ProRoot MTA (Dentsply Sirona Inc., Tulsa, Oklahoma) was placed as a retrograde filling material. Xenograft (Straumann Holding AG, Basel, Switzerland) was used to fill the debrided periapical region for bone regeneration and better structural stability (Figures 8, 9).

**Figure 10:**
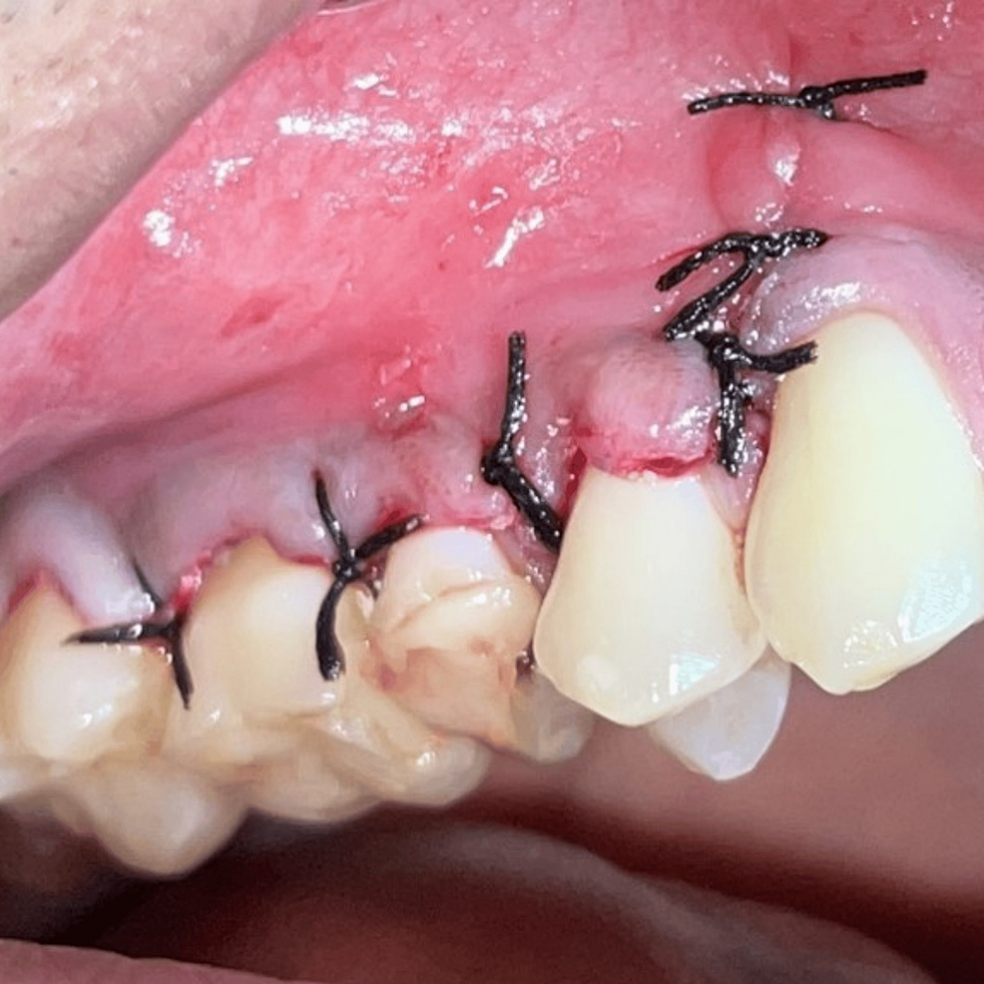
Flap advanced coronally to cover the recession in #14 and sutured

Finally, the flap was coronally advanced to cover the recession in relation to #14, and 3-0 non-resorbable sterile silk sutures (SC163, Unik Surgical Sutures, Unik, Taiwan) were placed (Figure 10).

Post-operative medications were prescribed after food: antibiotic amoxicillin 500 mg and metronidazole 400mg thrice daily for five days, and analgesic ibuprofen 400mg thrice daily for five days.

**Figure 11:**
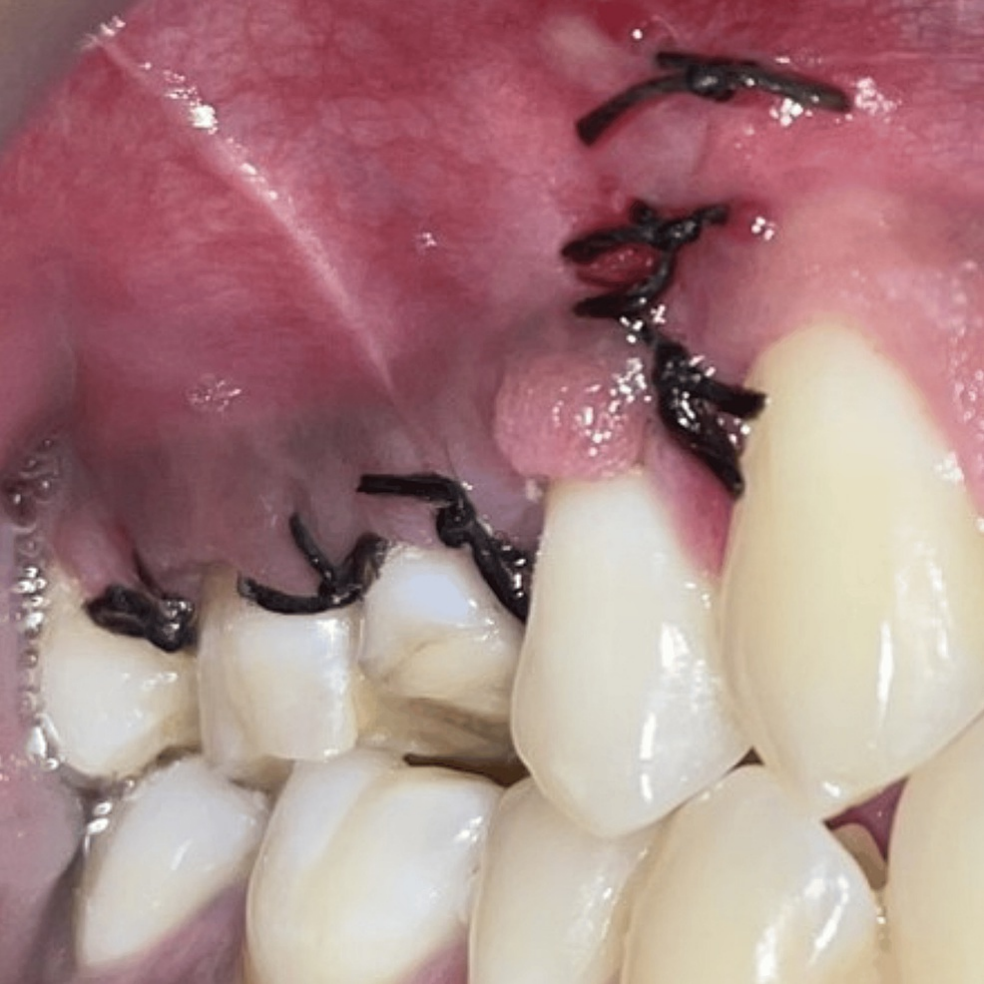
Two weeks post-surgery follow-up before suture removal

Post-surgery, the patient was recalled after two weeks for suture removal, and the healing appeared satisfactory (Figure 11).

**Figure 12:**
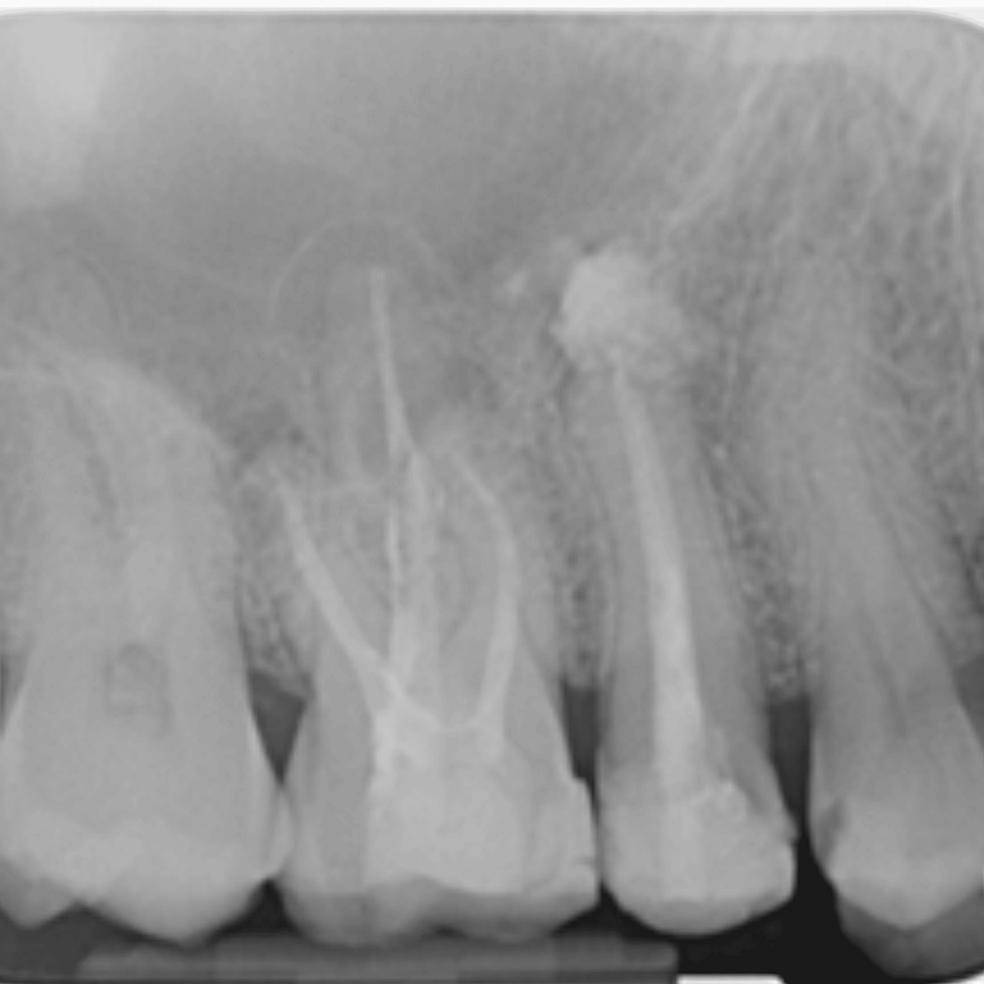
Six months postoperative IOPA IOPA: intraoral periapical radiography

A continuous review for six months was done to ensure the patient was asymptomatic in relation to #15. A follow-up periapical radiograph was taken for the patient to reconfirm the healing, which was satisfactory (Figure 12).

The post-space preparation was performed in the palatal canal with a post-drill (VDW GmBH, Munich, Germany) size 2, followed by fiber post-luting using resin cement (Relyx U200, 3M ESPE AG, Seefeld, Germany). Finally, a composite buildup was done, and the patient was referred to the Department of Prosthodontics for crown placement in relation to #15.

**Figure 13:**
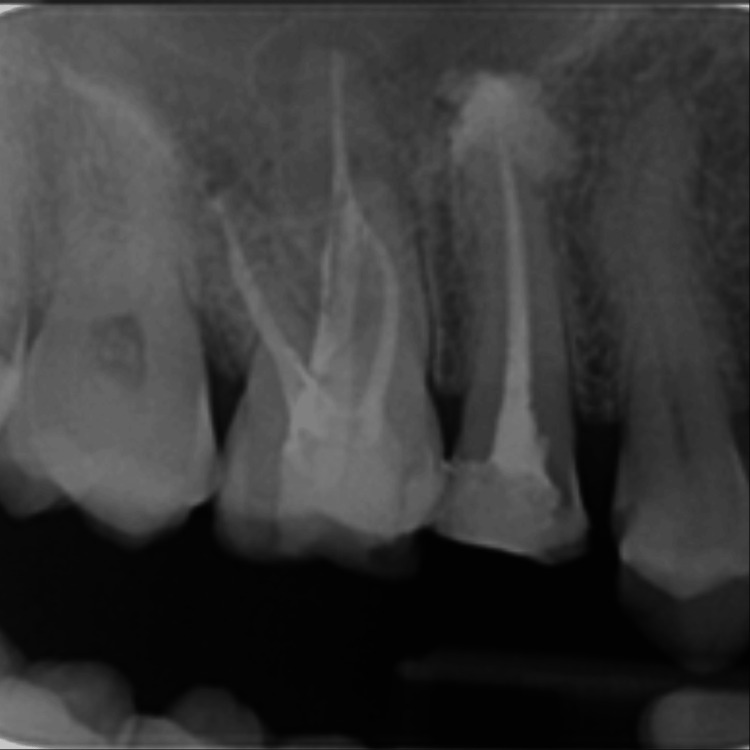
12-month follow-up IOPA before crown placement IOPA: intraoral periapical radiography

After 12 months, a periapical radiograph was taken for #15, and it showed complete healing in the periapical area, and good bone formation was observed (Figure 13).

**Figure 14:**
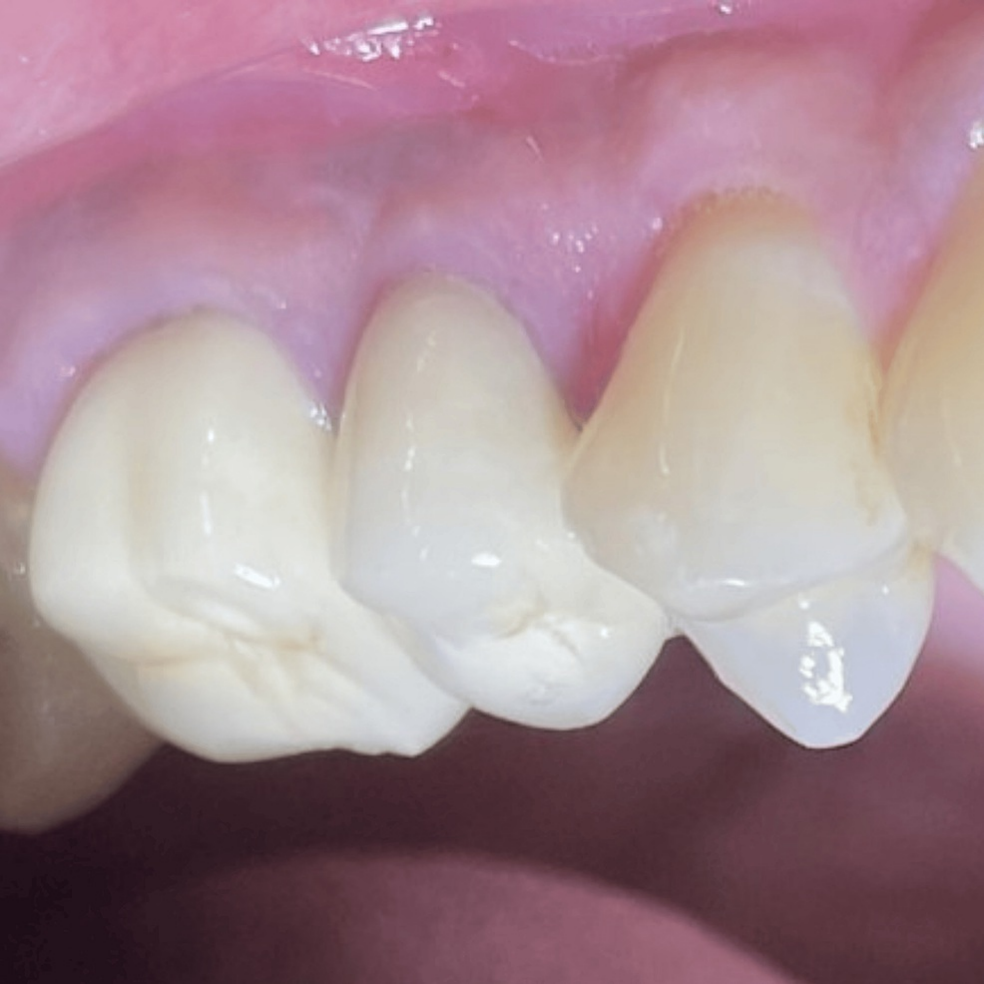
Gingival coverage in #14 and crown placement in #15 and #16 after 12 months

Gingival coverage in relation to #14 was seen during one year of follow-up and crown placement was done in relation to #15 and #16 (Figure 14).

## Discussion

The predominant cause of endodontic failure is persistent microbial infection [10]. The involvement of bacteria in peri-radicular infection has been well documented in the literature, and the chance of endodontic failure is more likely if microorganisms remain in the canals, defying root canal disinfection protocols following root canal obturation [11]. The harbored bacteria in the root canal complexities, such as isthmuses, dentinal tubules, and ramifications, also contribute to the failure of endodontic therapy [12].

Indication of surgical endodontic treatment for cases in which non-surgical treatment has been completed and the tooth continues to have periapical pathosis, which may or may not be symptomatic. The outcome was reported to have a success rate ranging from 27.84% to 80% [11].

When a periapical lesion is observed on a radiograph, clinicians are unaware of the histological status of the tooth at the time of treatment, and 10% of all periapical lesions end up requiring surgical intervention [13]. Surgical endodontics is the best approach for addressing failed re-treatment cases due to loss of apical seal and procedural errors, especially if post-endodontic restorations are present. The complex anatomy of the root canal often affects the success of nonsurgical endodontic therapy [14,15].

It has been observed that 98% of the apical ramifications and 93% of the lateral canals in the apical third are eliminated by resecting 3mm of the root during periapical surgery [16]. A resection angle perpendicular to the long axis of the root allows significant preservation of the apical root structure, increases the chance of identifying lingual and accessory canals, and enhances the coaxial root-end preparation with ultrasonics [17].

The ultrasonic retro-tips were used to prepare a 3mm root-end cavity that is centered and parallel to the long axis of the root to maintain adequate wall thickness and retain a biocompatible filling material [18]. Furthermore, removing isthmus tissue between two canals within the same root is possible, with a lower risk of damaging the surrounding soft tissues during the surgical procedure [19].

In this case report, a calcium silicate-based MTA (mineral trioxide aggregate) was employed for retrograde restoration, chosen for its remarkable biocompatibility and sealing prowess. Presently, calcium silicate cements are widely recognized as exceptional materials, owing to their bioactive and osteoconductive characteristics, and a high success rate with MTA as a root-end filling material was observed in a two- to six-year follow-up study [20].

## Conclusions

This case highlights the beneficial effects of incorporating bone grafting into endodontic surgical interventions for persistent periapical lesions. The results demonstrated favorable clinical and radiographic indicators of healing, which may significantly contribute to the successful treatment of similar cases. Therefore, performing apical surgery in conjunction with regeneration is a reliable choice to preserve the tooth and avoid more extensive measures like extraction of the tooth and its prosthetic replacement. The incorporation of ultrasonics, bone grafts, and biocompatible materials such as MTA enhances the overall prognosis. In conclusion, surgery serves as an excellent alternative to prevent tooth loss.
